# Predictive Value of C-Reactive Protein for Early Postoperative Complications in Children After Hypospadias Surgery

**DOI:** 10.3389/fped.2021.690863

**Published:** 2021-09-13

**Authors:** Fengming Ji, Haoyu Tang, Chengchuang Wu, Li Chen, Huake Wang, Bing Yan

**Affiliations:** ^1^Yunnan Key Laboratory of Children's Major Disease Research, Urology Department, Kunming Children's Hospital, Kunming, China; ^2^Yunnan Key Laboratory of Children's Major Disease Research, Department of General Surgery, Kunming Children's Hospital, Kunming, China

**Keywords:** hypospadias, C-reactive protein, complications, fistula, risk factor

## Abstract

**Background:** This study explored the predictive value of postoperative C-reactive protein in children with hypospadias for postoperative complications and the risk factors.

**Methods:** The clinical and follow-up data of 106 children with hypospadias who were treated with operations at Kunming Children's Hospital in 2020 were, respectively, analyzed. According to the occurrence of postoperative complications, the patients were divided into two groups: 25 patients with postoperative complications were the complications group, and 81 without postoperative complications were the control group. The baseline data, clinical characteristics, laboratory test indexes, and outcome of the two groups were collected. Receiver operating characteristic (ROC) was used to calculate the optimal cutoff value of C-reaction protein (CRP). Logistic regression was used to analyze the risk factors of hypospadias after surgery. A probability value (*P*) < 0.05 was considered statistically significant.

**Results:** According to the result of the ROC curve, the optimal cutoff value of CRP was 11.7 mg/L. Logistic regression showed that the length of urethral defect, the urethral material, the operative produce, and the postoperative CRP level were related to the occurrence of postoperative complications of patients with DCC. The length of the urethral defect and the CRP level were the independent risk factors of the prognosis of hypospadias patients. The CRP level was related to the occurrence of postoperative complications and fistula.

**Conclusions:** Postoperative CRP level can be used as a reliable marker for predicting the prognosis of hypospadias patients.

## Background

Hypospadias is a common congenital malformation in male individuals, which is mainly manifested by an abnormal ventral opening of the urethral meatus, abnormal distribution of foreskin, and chordee. The incidence of hypospadias is about 1/300–1/250 (1, 2). In the past 30 years, the incidence of hypospadias has been on the rise (3, 4). Heredity and gene mutations are common causes of hypospadias, and mutations of androgen receptor, Wilms tumor protein-1, steroid-α reductase, and other genes are closely related to the occurrence of hypospadias. Moreover, ecological environmental pollution, maternal exposure history of estrogen and antiepileptic drugs during pregnancy, maternal history of hypertension or hepatitis B virus infection, multiple births, and placental insufficiency are also potential risk factors for hypospadias (1, 5, 6).

Surgery is the only cure for hypospadias, and the repair of hypospadias aims to create a straight penis, a neourethra with a meatus at the tip of glans, and a normal appearance of a circumcised phallus (7, 8). However, long-term follow-up studies have found that adults with hypospadias may have abnormal urethral function, unsatisfactory appearance of the penis, and problems with erection, ejaculation, or sexual intercourse, which often negatively affect their quality of life (9). Many people with hypospadias need psychological treatment as adults. Improving the operative success rate is the key to cure genitalia malformations and meet the cosmetic needs of patients.

After more than 100 years of accumulation and evolution, there are more than 300 kinds of hypospadias surgical methods (10). However, due to the delicate and complex operation and the high technical difficulty of the operation itself, restricted by own conditions of the patient, the success rate after hypospadias is still low at present. Nearly 15–50% of patients need repeated operations, which not only causes a waste of medical resources but also seriously affects the appearance and function of the external genitalia of the patient' (11, 12). Therefore, how to improve the success rate of hypospadias surgery is still an important challenge for urologists for a long time.

In recent years, inflammatory markers have played an important role in the study of various postoperative complications. The acute-phase protein C-reactive protein (CRP) is a key factor in the clearance of bacteria and dying cells, so CRP is an important biomarker for inflammation (13). We hypothesized that CRP is associated with surgical outcomes in patients with hypospadias. Elevated CRP leads to an increased incidence of postoperative complications. The aim of this study was to confirm the relationship between postoperative complications of hypospadias and CRP.

## Materials and Methods

### Patients and Treatment

A retrospective database on patients with a primary hypospadias repair at Kunming Children's Hospital was performed in 2020. All the children included in the study signed the relevant informed consent before surgery. According to the location of the ventral opening, the degree of curvature, the condition of the urethral plate, and the distribution of foreskin, the operation produced was selected. All operations were performed by the same experienced surgeon. Blood routine and CRP tests were performed on the first day after surgery. Cefazolin sodium pentahydrate was routinely given half an hour before surgery and 5–7 days after surgery as prophylactic antibiotics (14). If surgical site infections occur, the duration of antibiotic use and the types of antibiotics were extended according to the infection situation. All patients were routinely indwelling an F6 or F8 all-silicone urethral catheter after the surgery, and the catheter was removed 10–30 days after the operation. The diameter of the urethral stent was according to the condition of the children.

### Inclusion and Exclusion Criteria

All blood samples were obtained 24 h after surgery. The inclusion criteria of this study were as follows: (1) The clinical information of patients were complete; (2) Follow-up data were obtained for all patients; and (3) Patients only underwent urethral repair. The exclusion criteria included the following conditions: (1) the clinical information of the patients was incomplete, (2) patients who underwent urethral repair and other surgery (undescended testis or inguinal hernia), (3) patients were lost to follow-up.

### Follow-Up

All the patients were followed up by outpatient consultation and telephone, and the follow-up deadline was February 1, 2021. The complications included overall complications, fistula, stricture, diverticulum, and glans split.

### Methods

The optimal cutoff values of CRP and WBC were determined by ROC curves. Enumeration data were expressed as median and interquartile range (IQR). The analysis of risk factors for overall complications adopted univariate and multivariate binary logistic regression analysis, and the results were expressed as a hazard ratio with 95% confidence interval (95% CI). *t*-test was used to analyze the correlation between CRP and complications. *P* < 0.05 was considered a statistically significant difference. The statistical analyses were performed with the Statistical Package of the Social Sciences (SPSS), version 21.0.

## Results

### General Information and Follow-Up Results

A total of 106 children were included in this study (the general information of the patients is shown in Table 1). The median follow-up was 6 months (IQR: 5–10). There were 25 cases in the complications group and 81 cases in the control group. The median age was 42 months (IQR: 35–58). The hypospadias classification included were 37 proximal, 47 middle, and 22 distal, respectively. The surgical procedures included MAGPI, tubularized incised urethral plate urethroplasty (TIP), Onlay island flap urethroplasty (Onlay), Koyanagi, two-stage urethroplasty, and Duplay (the general information of the patients and the results of the binary logistic regression univariate analysis are shown in Table 1). A total of 25 children had postoperative complications. Among all complications, the most common one was fistula with 16 on 25 complications. A total of 81 patients were cured, and the overall operation success rate was 76.42% at a median follow-up of 6 months.

**Table 1:** The results of binary logistic regression univariate analysis.

**Variates**	**Complication**	**No complication**	**Total**	***P***	**95% CI**
Age	46 (IQR:34~56)	41 (IQR:35~58)	41 (IQR:35~58)	0.973	0.979~1.022
**History of prematurity**					
Yes	6	17	23	0.072	0.295~2.473
No	19	6	25		
**Low birth weight infant**					
Yes	11	20	31	0.073	0.166~1.084
No	14	61	75		
**One of the twins**					
Yes	4	5	9	0.142	0.086~1.421
No	21	76	97		
History of prenatal estrogen use	8	18	26	0.339	0.229~1.662
	17	63	80		
**Grade of hypospadias**					
Distal	13	24		0.051	0.582~4.002
Middle	11	31			
Proximal	1	26			
**Chordee**					
≤ 15°	6	38		0.129	0.791~8.047
15°~30°	10	21			
≥30°	9	22			
Operating time	115 (IQR:105~133.75)	100 (IQR:80~125)	105 (IQR:88.75~130)	0.190	0.976~1.005
The length of urethral defect	23.5 (IQR:2~4.25)	2 (IQR:1~3.5)	2 (IQR:1.5~4)	0.006	0.489~0.884
**Urethral skin flap**					
In-suit flap	7	53	60	0.004	1.598~10.890
Graft flap	18	28	46		
**Diameter of urethral stent**					
F6	12	34	46	0.793	0.360~2.180
F8	13	47	60		
**Surgical procedure**					
MAGPI	1	18	19	0.009	0.007~0.010
TIP	3	30	33		
Onlay	7	7	14		
Koyanagi	2	3	5		
Ducktett	8	17	25		
STU	3	5	8		
Duplay	1	1	2		
**CRP**					
≤ 11.70	3	62	65	<0.001	0.011~0.158
>11.70	22	19	41		
**WBC**					
≤ 8.74	5	33	38	0.060	0.957~8.240
>8.74	20	48	68		

### The Predictive Value of CRP and WBC for Postoperative Complications

**Figure 1:**
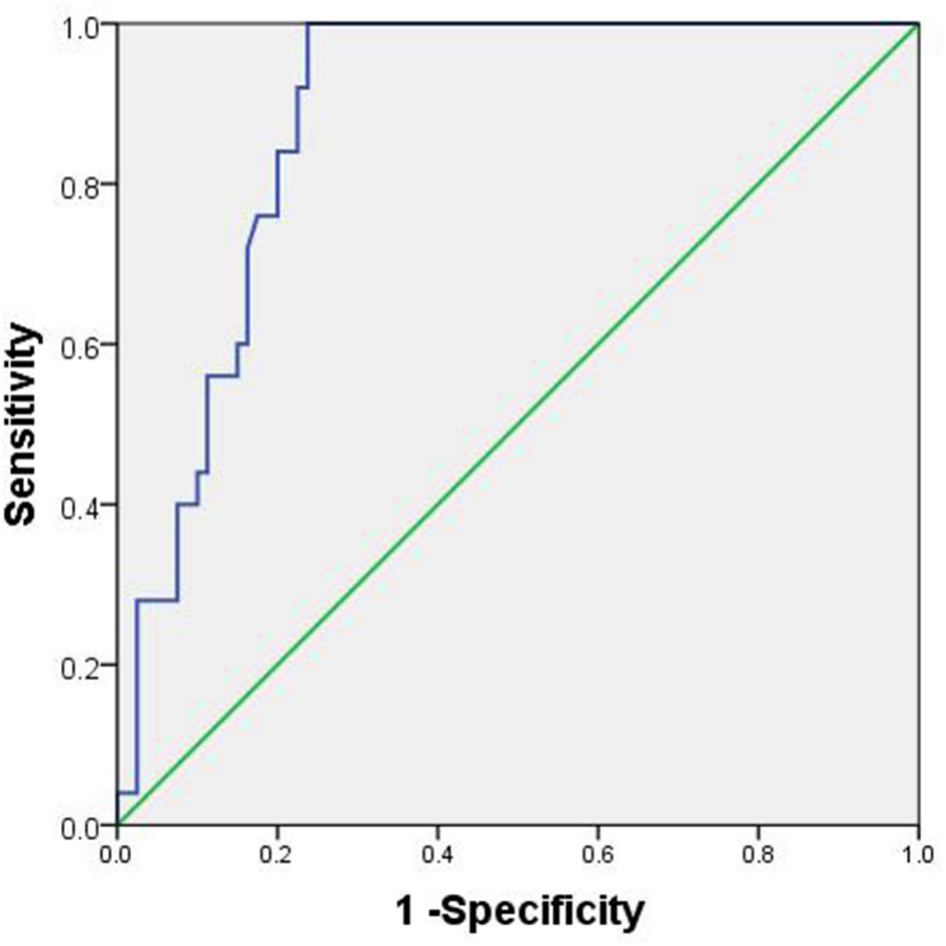
ROC curve of CRP.

According to the ROC curve analysis, the optimal cutoff value of CRP was 11.70 for predicting postoperative overall complication. The area under ROC curve for survival status was 0.882, with 95% CI of 0.820–0.944 (*P* < 0.001). The sensitivity and the specificity of CRP for hypospadias were 100 and 76.2%, respectively (Figure 1).

**Figure 2:**
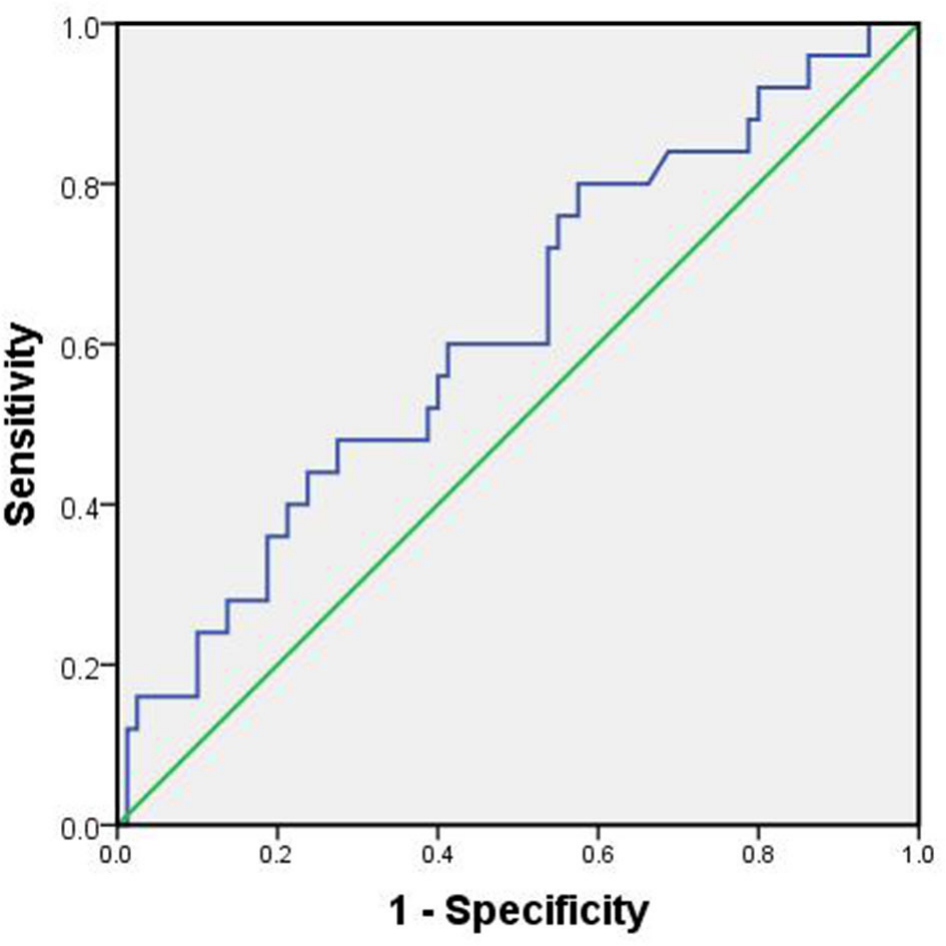
ROC curve of WBC.

The optimal cutoff value of WBC was 8.74 for predicting postoperative overall complication. The area under ROC curve for survival status was 0.620, with 95% CI of 0.493–0.0.747 (*P* = 0.071). The sensitivity and the specificity of WBC for hypospadias were 80 and 42.5%, respectively (Figure 2).

### Risk Factor Analysis Results

A binary logistic regression model was used to find the risk factors for the overall complications, and the analyzed factors include age, operative time, the length of urethral defect, urethral material (in-suit flap or graft flap from foreskin or scrotum), the diameter of the urethral stent, surgical procedure, CRP, and WBC. The results showed that the length of the urethral defect (*P* = 0.006), the resource of urethral skin flap (*P* = 0.004), the surgical procedure (*P* = 0.009), and CRP (*P* < 0.001) were the risk factors affecting the success rate of hypospadias. Among the four risk factors, urethral material and CRP (<0.001) were independent risk factors for the overall complications (the result of the binary logistic regression model is shown in Table 2).

**Table 2:** The result of binary logistic regression model.

**Variates**	**β**	**BE**	***P***	**95% CI**
The length of urethral defect	0.003	0.422	0.995	0.438~2.295
Urethral skin flap (In-suit flap)	2.539	0.885	0.032	5.728~8.364
Surgical procedure (Onlay)	0.876	2.067	0.672	0.042~138.150
CRP (<11.70)	3.147	0.761	<0.001	5.237~103.280

An independent-sample *t*-test was used to analyze the correlation between CRP value and overall complications, fistula, stricture, diverticulum, and glans split. The results showed that CRP was associated with overall complications (*P* < 0.001) and urethral fistula (*P* < 0.001), and the difference was statistically significant (the relationship between CRP and clinical characteristics in hypospadias is shown in Table 3).

**Table 3:** Relationship between CRP and clinical characteristics in hypospadias.

		**CRP ≤ 11.7**	**CRP > 11.7**	***T***	***P***
Age		50.84 ± 22.84	44.66 ± 18.03	1.46	0.145
Operating time		104.61 ± 35.15	113.29 ± 23.65	−1.52	0.132
The length of urethral defect		2.14 ± 1.55	3.05 ± 1.52	−2.89	0.005
Hypospadias classification	Proximal Middle Distal	25 20 20	2 22 17	−2.97	0.004
Chordee	≤ 15° 15°~30° ≥30°	32 15 18	12 16 13	−1.45	0.151
Urethral skin flap	In-suit flap Graft flap	45 20	17 24	−2.91	0.004
Diameter of urethral stent	F6 F8	31 34	18 23	−0.378	0.706
Surgical procedure	MAGPI TIP Onlay Koyanagi Duckett STU Duplay	16 23 4 3 13 5 1	2 11 10 2 12 3 1	−2.045	0.043
Overall complications	Yes No	3 62	22 19	5.884	<0.001
Fistula	Yes No	2 63	14 27	3.971	0.001
Stricture	Yes No	1 64	6 35	2.253	0.008
Diverticulum	Yes No	0 65	2 39	1.432	0.076
Glans split.	Yes No	0 65	2 29	1.432	0.076

## Discussion

The repair of hypospadias was recorded by Galen in the first to second century AD. In 1994, Snodgrass successfully performed and reported the TIP procedure for the first time. Up to now, TIP has become the most commonly used technique for the treatment of proximal and middle hypospadias, and it is the most popular procedure in Europe (15, 16). In 1980 and 1987, Duckett successively performed the Duckett and Onlay surgical procedures, in which the newly formed urethra was composed entirely or with the involvement of the prepuce inner plate with a vascularized pedicle, which greatly improved the primary cure rate of patients with hypospadias (17, 18). Although the surgical methods of hypospadias are constantly improving and evolving, there is still no surgical procedure that can be used as the gold standard for all types of hypospadias. Common complications of hypospadias include fistula, stricture, diverticula, and glans split (19). Studies have pointed out that the age of the patient, the length of the urethral defect, chordee, glans width, surgical procedure, anesthesia method, the diameter of the urethral stent, and preoperative hormone use history are all related to the success rate of surgery (7, 20–22).

This study retrospectively analyzed the clinical and follow-up data of 106 children with hypospadias and found that the urethral skin flap and CRP, as independent risk factors, had a high predictive value for postoperative complications. The incidence of postoperative complications in patients with CRP > 11.7 on the first day after surgery was significantly higher than that in patients with CRP ≤ 11.7, and the sensitivity and the specificity were 100 and 76.2%, respectively. The role of CRP has attracted a lot of attention in recent years, and many studies have pointed out that CRP is closely related to the prognosis of surgical patients. Hoeller et al. (23) pointed out that CRP had a high predictive value for postoperative surgical site infection after dorsal spondylodesis, with sensitivity of 92.9% and specificity of 78.2%. Messies et al. (24) demonstrated that CRP ≥ 180 mg/L on the 4th day after surgery was an independent risk factor for predicting anastomotic fistula after primary anastomosis in patients with rectal cancer (*P* = 0.002), with sensitivity and specificity of 72.3 and 88.9%, respectively. Johnson et al. (25) found that postoperative CRP value was an independent risk factor affecting 1-year tumor-free survival (*P* < 0.001) and 1-year overall survival (*P* < 0.001) after nephrectomy in patients with local renal cancer.

CRP is an acute-phase protein that appears in the process of the acute inflammatory response of the body. The level of plasma CRP in healthy individuals is <1 mg/L (26). When an inflammatory response occurs in the body, interleukin-6 can stimulate the liver cells to produce and secrete CRP rapidly, so that plasma CRP level can rise rapidly within 24–72 h, and then CRP can be produced by vascular smooth muscle cells, adipocytes, and kidneys (27). Now that CRP is highly sensitive to bacterial infection and is not affected by gender, age, anemia, and other factors, CRP has a high diagnostic value for infection-related diseases (28). CRP is a member of the superfamily of pentraxins, and it consists of five identical, non-covalently attached subunits (29). The pentameric structure of CRP includes an effector surface with an affinity for C1q and Fcy receptors and a recognition surface, which can bind phosphoric acid to dead cells and pathogens as well as nuclear components exposed in the process of apoptosis to clear necrotic cells and pathogens and protect autoimmunity (30–32). The half-life of CRP in the circulation is very short, about 19 h, and decreases rapidly after the relief of the acute reaction. Therefore, CRP is widely used in clinical practice as an important marker of acute infection (33).

Urethral fistula is the most common postoperative complication of hypospadias, with an incidence of about 5–10% (34, 35). Urethral fistula is related to flap ischemia, infection, high tension of the shaped urethra, and improper or too thin urethral covering (36, 37). In this study, the CRP value on the first postoperative day was associated with the occurrence of a urethral fistula (*P* < 0.001), and both urethral skin flap and CRP were independent risk factors affecting the success rate of hypospadias surgery. The reason is that the blood supply and the nutrition of the migratory skin flap completely depended on the vessel pedicle at the early stage, and the new blood circulation system was gradually established for the skin flap about 3 weeks after the operation (37). In the process of dissection, the migration flap is prone to damage excessive nourishing vessels, leading to the fistula due to the ischemic necrosis of the flap and stimulating the increase of CRP in peripheral blood.

Due to the small sample size and short follow-up time included in the study, the surgical method and length of the urethral defect in this study did not have great predictive value, which was different from existing studies. Due to the short follow-up time, the rate of stricture and diverticulum appeared to be low, actual surgical success need to be time censored with further follow-up. WBC is highly sensitive to predict bacterial infection, but this study found that WBC value is not correlated with postoperative complications of hypospadias. It is considered that peripheral blood white blood cells have poor stability, and bacterial infection, trauma, and stress can lead to the increase of WBC. Secondly, this study is a retrospective study, with some deficiencies in the integrity and homogeneity of the sample data, and the results of the study inevitably have a certain degree of recall bias. Finally, other variables including weight and BMI, history of prematurity, IGR, weight at birth, grade of hypospadias may be other potential risk factors but were not analyzed in this study.

## Conclusion

In conclusion, the length of the urethral defect, urethral material, surgical method, and CRP are related to the prognosis of patients with hypospadias. Urethral material and postoperative CRP value are independent risk factors for the prognosis of patients with hypospadias. The CRP value is easy to obtain and cheap in practical clinical work. Routine test of CRP value after surgery is helpful for the early identification of high-risk patients. For the high-risk patients, intervention measures should be given timely, such as keeping on antibiotics for a longer time or keeping the catheter for more days, which has a great significance for improving the success rate of hypospadias surgery.

## Data Availability Statement

The original contributions presented in the study are included in the article/supplementary material, further inquiries can be directed to the corresponding authors.

## Ethics Statement

This study was approved by the Medical Ethics Committee of Kunming Children's Hospital.

## Author Contributions

FJ collected, analyzed data, and drafted the original manuscript. HT and LC collected data and participated in amending the manuscript. CW collected and analyzed data. HW analyzed data. BY designed the present study and amended the manuscript. All authors contributed to the article and approved the submitted version.

## Conflict of Interest

The authors declare that the research was conducted in the absence of any commercial or financial relationships that could be construed as a potential conflict of interest.

## Publisher's Note

All claims expressed in this article are solely those of the authors and do not necessarily represent those of their affiliated organizations, or those of the publisher, the editors and the reviewers. Any product that may be evaluated in this article, or claim that may be made by its manufacturer, is not guaranteed or endorsed by the publisher.
